# Unidirectional emission of GaN-on-Si microring laser and its on-chip integration

**DOI:** 10.1515/nanoph-2022-0577

**Published:** 2023-01-03

**Authors:** Hanru Zhao, Meixin Feng, Jianxun Liu, Xiujian Sun, Tao Tao, Qian Sun, Hui Yang

**Affiliations:** School of Nano-Tech and Nano-Bionics, University of Science and Technology of China, Hefei 230026, China; Key Laboratory of Nano-Devices and Applications, Suzhou Institute of Nano-Tech and Nano-Bionics (SINANO), Chinese Academy of Sciences (CAS), Suzhou 215123, China; Guangdong Institute of Semiconductor Micro-nano Manufacturing Technology, Foshan 528000, China; School of Electronic Science and Engineering, Nanjing University, Nanjing 210093, China

**Keywords:** GaN, microring laser, on-chip integration, unidirectional emission, waveguide

## Abstract

GaN-based microring lasers grown on Si are promising candidates for compact and efficient light sources in Si-based optoelectronic integration and optical interconnect due to their small footprints, low mode volume, low power consumption, and high modulation rate. However, the high symmetry of circular microcavity leads to isotropic emission, which not only reduces the light collection efficiency, but also affects other adjacent devices during data transmission. In this study, the unidirectional lasing emission of room-temperature current-injected GaN-based microring laser was realized by coating metal Ag on the microring sidewall and integrating a direct coupled waveguide. The light was efficaciously confined in the cavity and only emitted from the waveguide, which avoided optical signal crosstalk with other adjacent devices. Furthermore, we integrated a microdisk at the other end of the waveguide as a photodetector, which could effectively detect the output power of the microring laser from the direct coupled waveguide. Therefore, a preliminary on-chip integration of GaN-based microring laser, waveguide and photodetector on Si substrate was successfully demonstrated for the first time, opening up a new way for on-chip integration and optical interconnect on a GaN-on-Si platform.

## 1 Introduction

III-nitride semiconductors with a direct bandgap, a wide spectral range from the deep ultraviolet to infrared, and high luminous efficiency, are excellent optoelectronic materials, which have been widely applied for light-emitting diodes (LEDs), laser diodes (LDs), and photodetectors [1, 2]. The outstanding luminous performance of III-nitrides is complementary to the shortcoming of Si, which with an indirect bandgap can’t emit light efficiently, offering a III-nitride-on-silicon platform for integrated photonics [3, 4]. GaN-based microdisk/microring LDs grown on cost-effective Si substrates are promising candidates as compact and efficient laser sources for the integrated photonics due to their small footprints, in-plane light coupling, low mode volume, low power consumption, and high modulation rate [5, 6], as well as have attracted great interest and been studied extensively for diverse applications, such as ultralow threshold laser sources [7], nonlinear optics [8], visible light communication [9], and on-chip optical interconnects [10].

However, the highly rotational symmetry of circular microcavity LDs usually leads to in-plane isotropic emission and hence inefficient light collection [11]. Besides, this isotropic light also interferes with other adjacent devices, which is not beneficial to optical signal transmission and limits their applications [12, 13]. Many research works are devoted to various device designs to realize the unidirectional emission by changing the radial symmetry of circular microcavity, such as eccentric microring structure [12, 14], spiral-shaped microcavity [15, 16], microdisk with a corner or a notch [17, 18], and etc. Although these deformed cavity designs can achieve the directionality of emission, further light-coupling techniques are still required for extracting and collecting efficiently the light. Direct coupled waveguide is a promising solution of realizing directional emission and efficient light collection at the same time [19–21].

Except as the above-mentioned excellent light sources, GaN-based quantum well diodes can also be used as photodetectors (PDs) to detect their output power, since their emission spectra overlap with the response spectra due to the same active region [22, 23]. Recently, on-chip integration of the GaN-based LED, coupled waveguide and photodetector on Si platform has been reported for multidimension spatial light communication and on-chip optical interconnect [24, 25]. Preliminary integration of the GaN-based LD with Fabry–Pérot cavity, modulator and photodetector on Si was also demonstrated [26]. As compared with GaN-based LED and LD with Fabry–Pérot cavity, GaN-based microring LDs have a smaller size, much higher modulation frequency, and more efficient coupling, which make them better suited as on-chip light sources. However, few literatures have reported the on-chip integration of GaN-on-Si microring lasers due to the big challenge for the simultaneous realization of unidirectional emission and efficient light coupling.

In this letter, by coating metal Ag on the microring sidewall and integrating a direct coupled waveguide at the microring rim, we achieved unidirectional emission of room-temperature current-injected GaN-on-Si microring laser, which effectively avoided the optical crosstalk with adjacent devices. Moreover, we also integrated a microdisk photodetector at the other end of the waveguide, and firstly demonstrated a preliminary on-chip integration of the microring laser, waveguide, and photodetector on a GaN-on-Si platform.

## 2 Experiment

**Figure 1:**
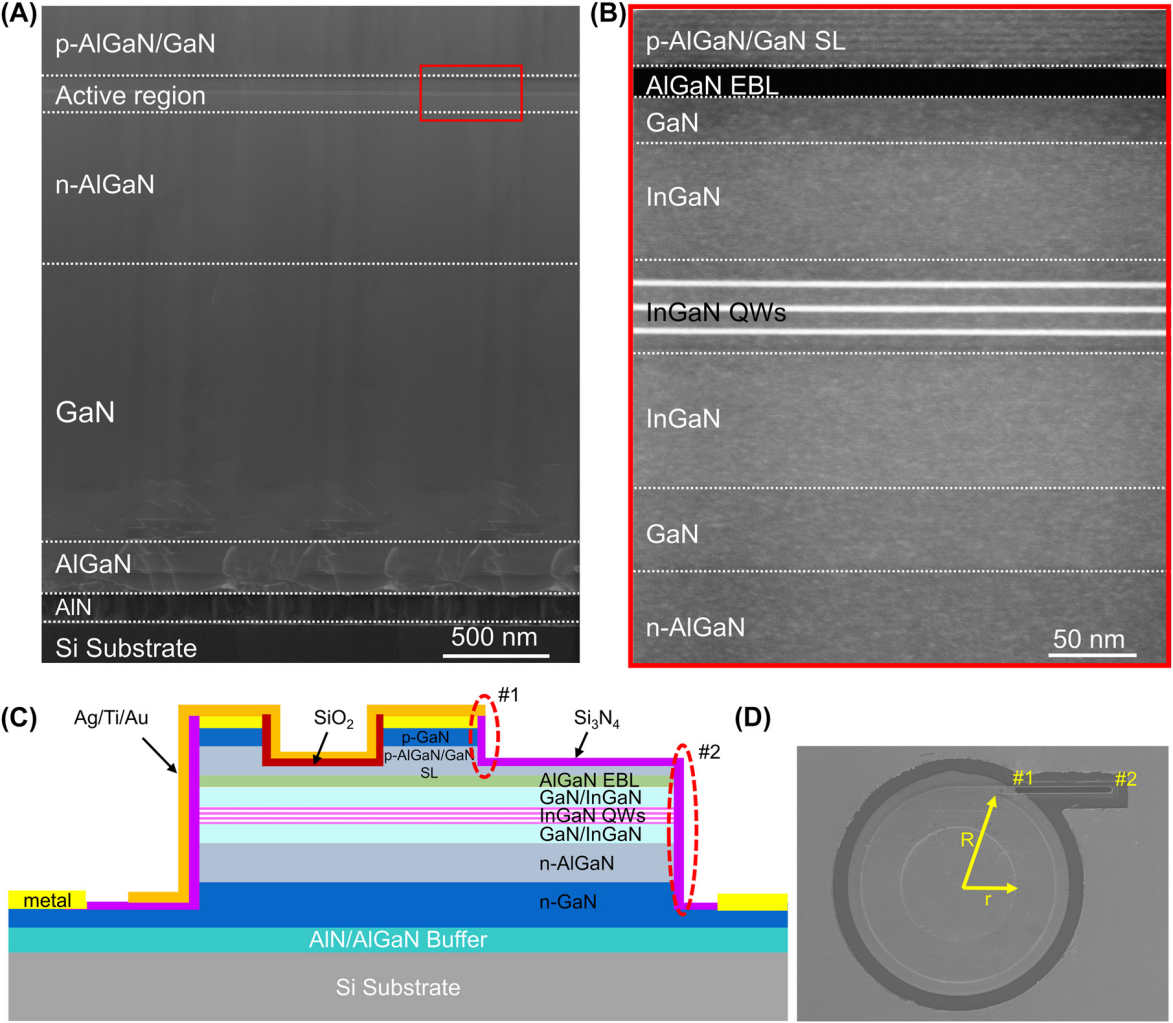
Epitaxial and schematic structures of GaN-on-Si microring lasers. (A) Cross-sectional STEM image of GaN-based microring laser epitaxial structure grown on Si (111) substrate. (B) Enlarged image of the marked red rectangle in (A). (C) Cross-sectional schematic diagram, and (D) top-view SEM image of the as-fabricated GaN-on-Si LD-W-Ag with an outer (*R*) and inner (*r*) radius of 50 and 30 μm, respectively. The points #1 and #2 in the figure are two end facets of the waveguide fabricated by etching.

Figure 1(A) and (B) show the cross-sectional scanning transmission electron microscope (STEM) image of the GaN-based microring laser epitaxial structure, sharing the same layers with the direct coupled waveguide and photodetector. To obtain high-quality GaN films on Si, an AlN/AlGaN multilayer buffer was firstly grown on Si substrate, to effectively compensate the tensile stress and prohibit the extension of threading dislocations (TDs) caused by the mismatches of thermal expansion coefficient and lattice between Si and GaN [27–29]. Afterwards, a 1.3 μm-thick unintentionally doped GaN layer, a 1.3 μm-thick n-type GaN contact layer, a 1.2 μm-thick n-type Al_0.085_Ga_0.915_N cladding layer, a 100 nm-thick In_0.01_Ga_0.99_N/GaN lower waveguide layer, three pairs of In_0.12_Ga_0.88_N/In_0.02_Ga_0.98_N quantum wells (QWs), a 90 nm-thick In_0.01_Ga_0.99_N/GaN upper waveguide layer, a 20 nm-thick p-type Al_0.18_Ga_0.82_N electron blocking layer (EBL), 600 nm-thick p-type Al_0.15_Ga_0.85_N/GaN superlattice (SL) cladding layers, and a 30 nm-thick p-type GaN contact layer were epitaxially grown in turn.

The as-grown GaN-on-Si microring laser epitaxial wafer was subsequently processed into microring LDs through a series of processing technologies. Firstly, Pd/Pt/Au (30/30/50 nm) were evaporated by electron-beam evaporation on the p-contact layer, followed by a rapid thermal annealing under air ambient at 600 °C for 90 s in order to form an ohmic contact. Subsequently, photolithography and ion beam etching were used to pattern and form an inner ring to avoid undesired optical absorption and heat generation thus reducing lasing threshold [30, 31], as well as remove the contact metal over the waveguide region to avoid unnecessary current injection, then a SiO_2_ insulation layer was deposited by inductively coupled plasma chemical vapor deposition (ICP-CVD) to prohibit current leakage. Next, GaN-based microring LDs coupled with a 3 μm-wide waveguide were formed with 500 nm Ni as mask by photolithography and dry etching down to n-GaN contact layer. Afterwards, to prevent current leakage, a 240 nm-thick Si_3_N_4_ dielectric layer was deposited on the microring sidewall by plasma-enhanced chemical vapor deposition after being treated in a 25% tetramethyl ammonium hydroxide (TMAH) solution at 85 °C for 20 min to remove the etching damage and shape the device sidewalls. After removing the Ni mask by using dilute hydrochloric acid, 300 nm-thick Ag was coated on both the sidewall and top surface of the microring LDs by magnetron sputtering, followed by the deposition of Ti/Au (80/110 nm) to prevent Ag oxidation. Finally, n-electrode was formed by evaporating Ti/Pt/Au (50/100/100 nm) without rapid thermal annealing. For comparison, GaN-on-Si microring LDs without Ag coating were also fabricated simultaneously.

For simplicity, the as-fabricated GaN-on-Si microring LDs covered with and without Ag are labeled as LD-W-Ag and LD-W/O-Ag, respectively. Figure 1(C) and (D) show the cross-sectional schematic diagram and top-view scanning electron microscopy (SEM) image of the as-fabricated GaN-on-Si LD-W-Ag, respectively. By depositing an insulating layer and Ag with high reflectivity in visible light around the microring, it is expected that the light can be effectively confined in the microring and only emitted from the direct coupled waveguide.

All the characterizations of GaN-on-Si microring LDs were performed at room temperature. The cross-sectional images of GaN-on-Si microring laser epitaxial structure were obtained by focused ion beam (FEI Scios) and STEM (FEI Talos F200×). The top-view and side-view images of the devices were observed by SEM equipment (Quanta 400 FEG) and optical microscope (Nikon DS-Fi2-U3), respectively. And the top-view luminous images were captured by a charge coupled device (CCD) camera. The electroluminescence (EL) spectra and light output power under current injection with a pulse width of 400 ns and a repetition rate of 10 kHz (supplied by Agilent 8114A Pulse Generator), were collected along the direction of the direct coupled waveguide by a high-resolution spectrometer (Ocean Optics HR 4000) and optical power meter (Thorlabs PM121D), respectively. The sampling port was slightly tilted to align with the waveguide end, and about 2 mm away from the light-emitting devices. The current–voltage (I–V) characteristics of the microring and the induced photocurrent of microdisk PD were measured by a continuous-wave power supply (Keithley 2400). The capacitance–voltage (C–V) characteristic was measured by a semiconductor parameter meter (Keithley 4200).

## 3 Results and discussion

**Figure 2:**
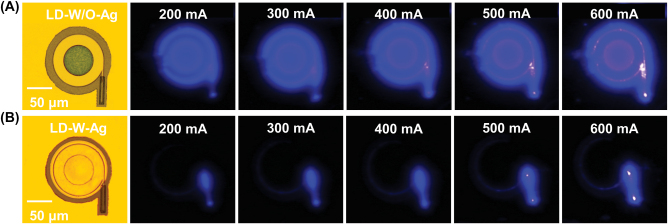
Top-view optical microscope and luminous images of GaN-on-Si microring lasers (*R* = 50 μm, *r* = 30 μm) at various pulse injection currents. (A) LD-W/O-Ag, and the left part of the emission patteren was blocked by the electrical probe. (B) LD-W-Ag. There were two emission points on the direct-coupled waveguide corresponding to points #1 and #2 in Figure 1(C) and (D), respectively. For the emission from point #1, it was mainly due to the height difference between microring and coupled waveguide caused by removing the metal on the waveguide.

Figure 2 shows top-view optical microscope and luminous images of LD-W-Ag and LD-W/O-Ag under pulsed injection currents from 200 to 600 mA, respectively. The luminescence of both LD-W/O-Ag and LD-W-Ag became stronger with the increase of injection currents. However, an apparent difference in the light emitting direction could be observed, the whole profile of LD-W/O-Ag still emitted light uniformly around the circular cavity, although there was a direct coupled waveguide, as shown in Figure 2(A). In contrast, the light emission of LD-W-Ag outputted only from the coupled waveguide, and the light around the microring was efficaciously confined as shown in Figure 2(B). These results indicated that we successfully realized the unidirectional emission of GaN-on-Si microring LD and effectively avoided the optical crosstalk with adjacent devices on a single chip by coating metal Ag on the microring sidewall and integrating a direct coupled waveguide.

**Figure 3:**
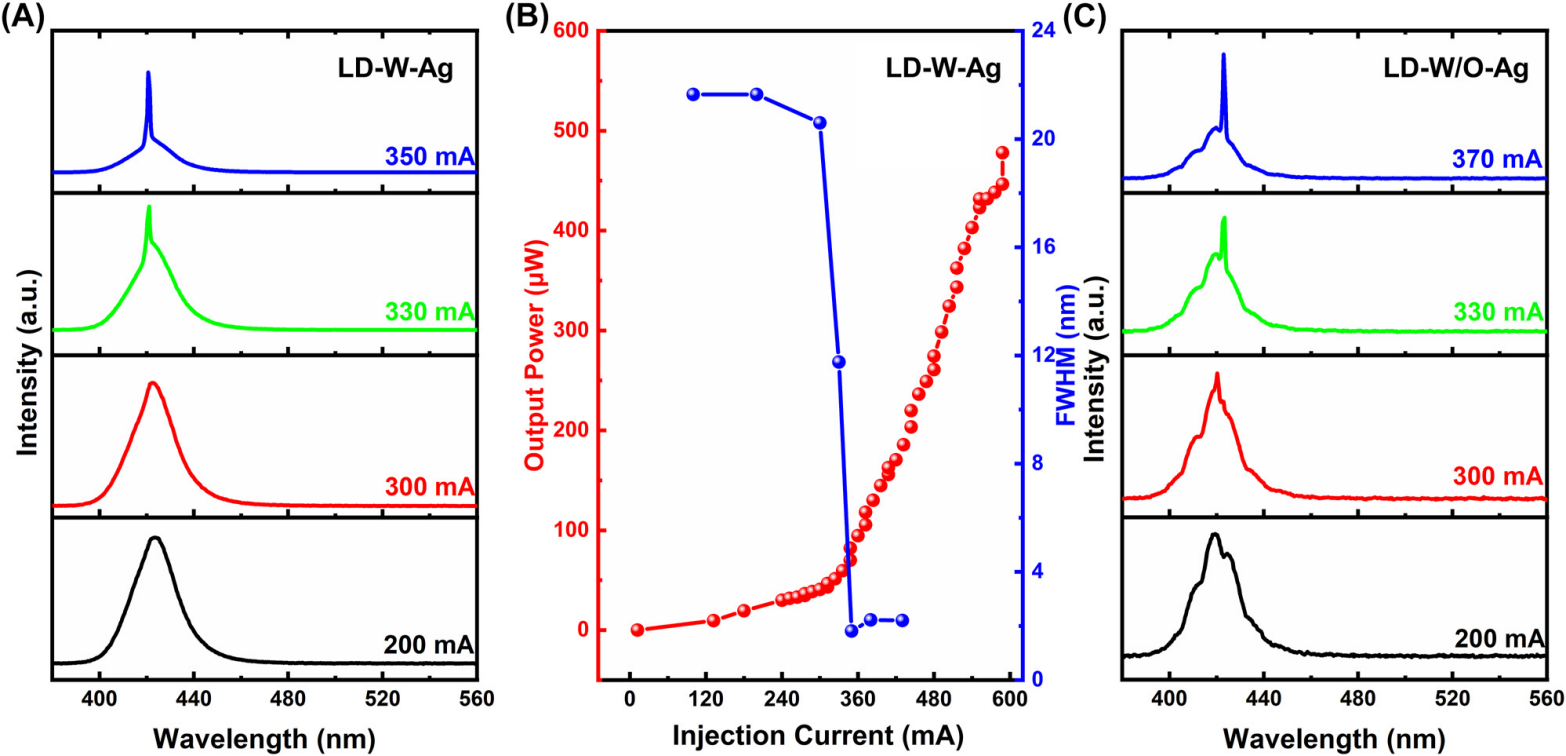
Luminescence performance of GaN-on-Si microring lasers (*R* = 50 μm, *r* = 30 μm). (A) EL spectra of LD-W-Ag measured under various pulse injection currents, (B) FWHM and light output power of LD-W-Ag as a function of injection current. (C) EL spectra of LD-W/O-Ag measured under various pulse injection currents.

The introduction of metal Ag for the microring sidewall may lead to absorption loss. In order to analyze this effect on luminescence performance of as-fabricated GaN-based microring LDs, EL characteristics of both LD-W-Ag and LD-W/O-Ag were measured along the direct coupled waveguide direction under various pulse injection currents at room temperature. As shown in Figure 3(A), broad EL spectra with a full width at half maximum (FWHM) of over 21 nm at a low injection current (<300 mA) were collected owing to strong spontaneous emission. When the injection current increased to 330 mA, there was a sharp emission peak appearing on the EL spectrum. As the injection current further increased to 350 mA, the stimulated emission was dominant, and the FWHM quickly narrowed down to 1.8 nm. Figure 3(B) clearly demonstrates the FWHM and output power as a function of injection currents. The output power increased slowly below the injection current of 350 mA, however, it increased abruptly when the injection current was larger than 350 mA. A clear turning point was also observed at 350 mA for the FWHM versus injection current curve. All of these observations indicated that the current injected lasing operation of the as-fabricated LD-W-Ag was achieved at room temperature. Moreover, EL spectra of the reference LD-W/O-Ag were also obtained as shown in Figure 3(C). The EL spectra presented a periodical oscillation pattern, attributed to the interference of vertical standing waves [32, 33], but still had a similar variation trend of the FWHM with that shown in Figure 3(A). When the operation current was less than 300 mA, broad EL spectra with a FWHM of about 20 nm were collected. With the injection current increasing, the FWHM of the EL spectrum narrowed down to about 10.2 nm at 330 mA and further reduced to about 1.7 nm at 370 mA. These results manifested that the introduction of Ag did not increase the lasing threshold of GaN-on-Si microring LD, and the detailed discussion will be in the following part.

**Figure 4:**
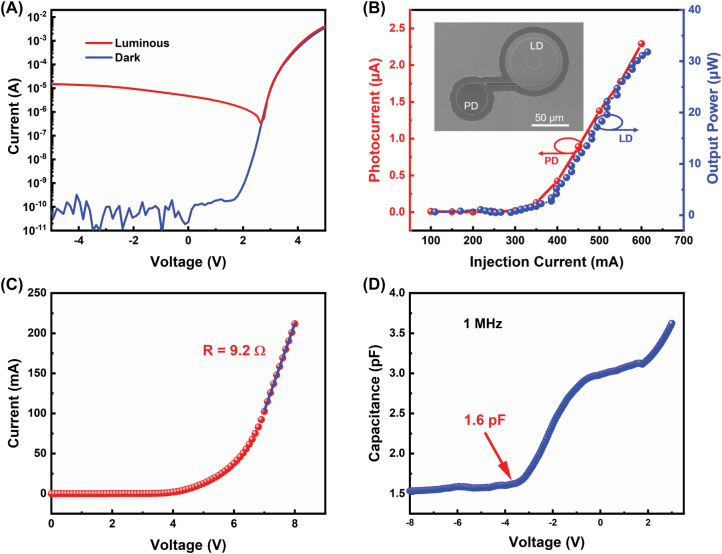
Characteristics of on-chip integration of the GaN-on-Si microring laser. (A) The measured I–V curves of a microdisk in dark and luminous of adjacent microring LD. (B) The induced photocurrent of a microdisk PD (*R* = 20 μm, *r* = 0 μm) without Ag coating and the light output power of a LD-W-Ag (*R* = 30 μm, *r* = 10 μm) as a function of the injection current at zero bias, the inset showed a bird-view SEM image of a preliminary on-chip integration of microring LD and microdisk PD directly connected by a 100 μm-long waveguide. (C) I–V characteristics of the LD-W-Ag (*R* = 30 μm, *r* = 10 μm), and (D) C–V measurement of the laser under a modulation frequency of 1 MHz.

To realize on-chip integration, the characteristic of GaN-based microdisk as a PD was examined, which was integrated with a microring LD by a direct coupled waveguide, as shown in the inset of Figure 4(B). Figure 4(A) shows the I–V curves of the as-fabricated microdisk without Ag coating as PD in the dark and luminous of the adjacent GaN-on-Si microring LD, respectively. It could be seen that at a reverse bias of −5 V, the reverse current of the microdisk in the dark was close to the measurement limit of the source meter (∼10^−11^ A), which proved the good fabrication process. However, the reverse current of the microdisk under the illumination of the adjacent microring LD at a direct current of 30 mA, sharply increased to about 1.5 × 10^−5^ A, six orders of magnitude higher than the dark current. This result clearly indicated that GaN-based microdisk could also be used as a PD. Based on this, the real-time monitoring of microring LD output power was performed by measuring the photocurrent of the PD. As presented in Figure 4(B), the measured photocurrent of the microdisk PD demonstrated an obvious threshold at the injection current of 350 mA, which was consistent with the measured output power versus injection current curve. All these observations manifested preliminary on-chip integration of GaN-based microring LD, coupled waveguide and PD on Si was performed successfully.

To evaluate the ability of data transmission for the as-fabricated microring laser, we calculated the resistor–capacitance (RC) limited bandwidth of the microring LD by measuring I–V and C–V curves. Figure 4(C) shows the I–V characteristic of the microring LD. The series resistance was calculated as 9.2 Ω, derived from the linear fitting of the measured I–V curve between 100 and 210 mA. The C–V curves was measured by varying the bias voltage from −8 to 3 V at a modulation frequency of 1 MHz. As shown in Figure 4(D), the capacitance of the microring LD was about 1.6 pF in a deep depletion state at bias voltage of −4.5 V. Thus, the RC time-constant of the as-fabricated microring LD was calculated to be ∼14.72 ps, corresponding to a RC-limited bandwidth of 10.8 GHz, which was promising to perform a large 3 dB modulation bandwidth and high modulation rate, showing great potential applications for high-speed in-plane data transmission and optical communication on a GaN-on-Si platform.

**Figure 5:**
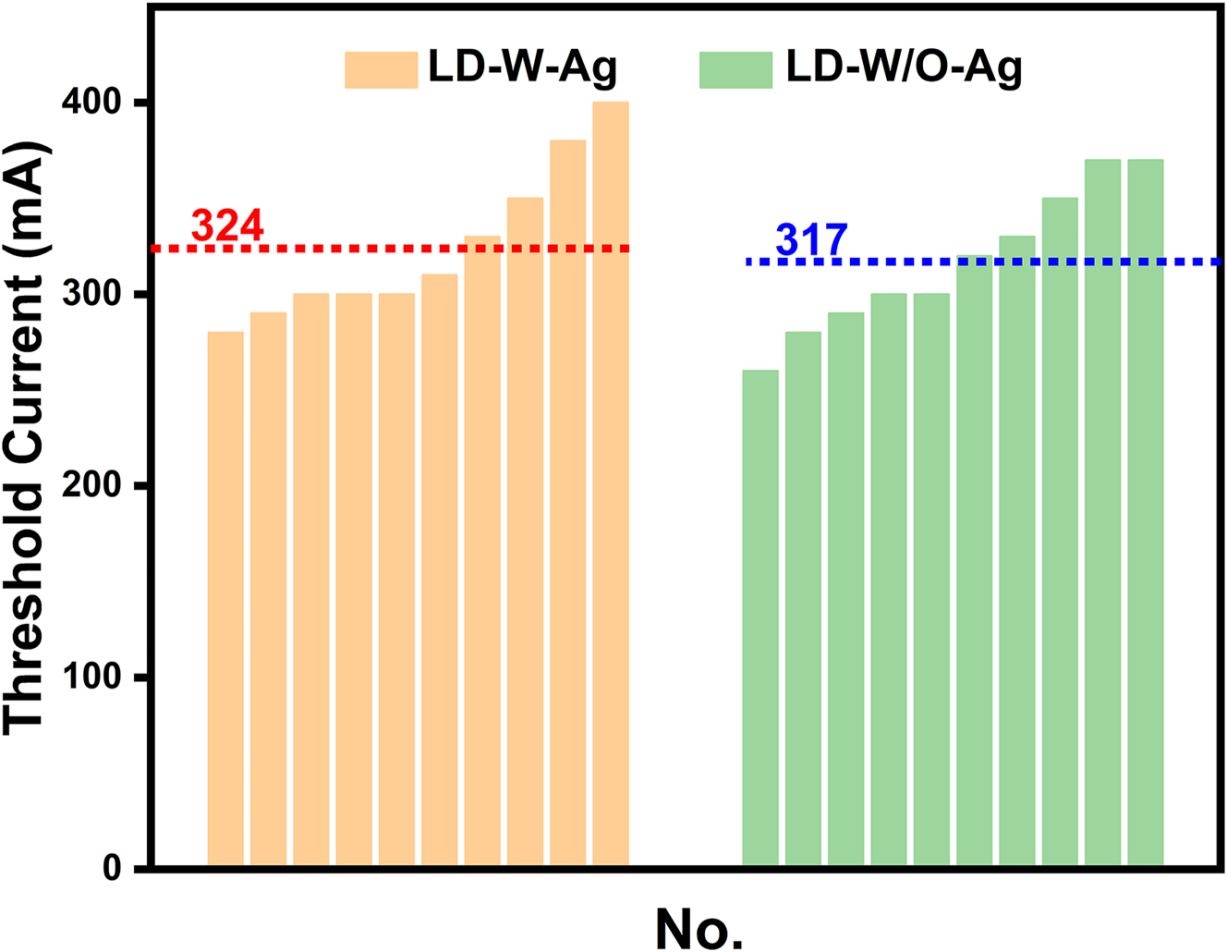
Threshold currents of LD-W-Ag and LD-W/O-Ag (*R* = 50 μm, *r* = 30 μm). The dot lines mean the average threshold currents of LD-W-Ag and LD-W/O-Ag.

**Figure 6:**
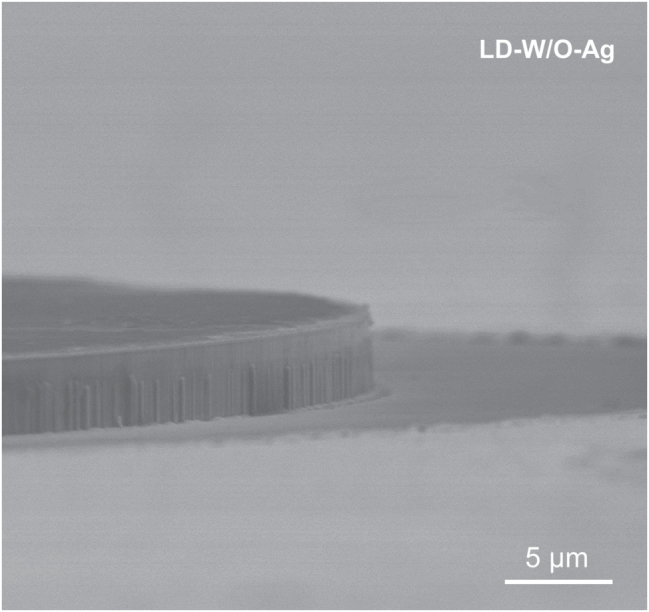
Side-view SEM image of the LD-W/O-Ag.

All these results indicated that by coating metal Ag on the microring sidewall and integrating a direct coupled waveguide, the light could be effectively confined in the cavity, as well as unidirectional lasing emission of the microring LD could be successfully realized without increasing the lasing threshold. As shown in Figure 3(A) and (C), the LD-W-Ag presented a similar lasing threshold with the LD-W/O-Ag, which was consistent with the statistic result displayed in Figure 5. It could be found that the lasing threshold currents of both devices varied with the range of 260–400 mA. But the average lasing threshold currents of LD-W-Ag and LD-W/O-Ag were almost equal, and the device thresholds near each other were similar. These results clearly demonstrated that the introduction of Ag layer didn’t significantly increase the lasing threshold of GaN-on-Si microring LD. On the one hand, the absorption loss of Ag increased the threshold by raising the total cavity loss; on the other hand, it reduced the threshold by suppressing high-loss mode operation and reducing mode competition. It could be seen that the EL spectra of LD-W-Ag performed fewer cavity modes as compared with those of LD-W/O-Ag, which was mainly due to that a mode-dependent loss deriving from metallic absorption suppressed multiple mode operation. A similar result has been reported by Xu et al. [34, 35], single mode lasing was demonstrated by placing GaN nanowire lasers onto a gold substrate, which induces a mode-dependent and polarization-sensitive loss and hence suppresses multiple transverse-mode operation. Therefore, the trade-off between these two processes results in an optimal lasing threshold, indicating that it is a feasible method to achieve unidirectional emission of microring laser by depositing a metal layer on the microring sidewall. At present, the as-fabricated GaN-on-Si microring LDs still presented a higher threshold current, which may be attributed to two reasons. One is the large scattering loss caused by unsmooth sidewall morphology owing to incomplete wet chemical polishing (20 min) as shown in Figure 6. Longer reaction time can produce steep and smooth sidewalls, but would destroy the 3 μm-wide waveguide. The other is the relatively high TD density due to huge mismatch in thermal expansion coefficient and lattice constant between GaN and Si. It has been shown that the internal quantum efficiency can be greatly enhanced by reducing the TD density in InGaN/GaN laser materials. Further improvement study of the optimization of the device fabrication process and the epitaxial growth of high-quality GaN-on-Si material, together with the reduction of the insulation thickness to enhance surface plasmon-enhanced effect and hence optical confinement is underway to improve the device performance [36, 37].

## 4 Conclusions

In summary, we carefully fabricated GaN-on-Si microring LD covered with metal Ag and outputted from a direct coupled waveguide. Subsequently, we characterized the EL performance of the as-fabricated microring LDs. The results showed that the light was laterally confined in the cavity and only emitted from the direct coupled waveguide, avoiding effectively the optical crosstalk with other adjacent devices during optical signal transmission on a single chip. And the GaN-on-Si microring LD realized room-temperature electrically pumped lasing with a similar threshold current as compared with the conventional microring LD without Ag coating. Afterwards, we integrated another microdisk at the other end of the coupled waveguide as PD, and firstly demonstrated the preliminary on-chip integration of GaN-based microring LD, waveguide and PD, showing great potential applications for high-speed in-plane data transmission and optical communication on a GaN-on-Si platform.

## References

[1] Cai W., Gao X., Yuan W. (2016). Integrated p–n junction InGaN/GaN multiple-quantum-well devices with diverse functionalities. Appl. Phys. Express.

[2] Xie M., Jiang Y., Gao X. (2021). Uniting a III-nitride transmitter, waveguide, modulator, and receiver on a single chip. Adv. Eng. Mater..

[3] Sellés J., Crepel V., Roland I. (2016). III-Nitride-on-silicon microdisk lasers from the blue to the deep ultra-violet. Appl. Phys. Lett..

[4] Tabataba-Vakili F., Rennesson S., Damilano B. (2019). III-nitride on silicon electrically injected microrings for nanophotonic circuits. Opt. Express.

[5] Mei Y., Xie M., Long H., Ying L., Zhang B. (2022). Low threshold GaN-based microdisk lasers on silicon with high Q factor. J. Lightwave Technol..

[6] Tang Y., Feng M., Zhao H. (2022). Electrically injected GaN-on-Si blue microdisk laser diodes. Opt. Express.

[7] Zhao C., Tang C. W., Wang J., Lau K. M. (2020). Ultra-low threshold green InGaN quantum dot microdisk lasers grown on silicon. Appl. Phys. Lett..

[8] Roland I., Gromovyi M., Zeng Y. (2016). Phase-matched second harmonic generation with on-chip GaN-on-Si microdisks. Sci. Rep..

[9] Shi Z., Gao X., Yuan J. (2017). Transferrable monolithic III-nitride photonic circuit for multifunctional optoelectronics. Appl. Phys. Lett..

[10] Thubthimthong B., Sasaki T., Hane K. (2015). Asymmetrically and vertically coupled hybrid Si/GaN microring resonators for on-chip optical interconnects. IEEE Photonics J..

[11] Kryzhanovskaya N., Zhukov A., Moiseev E., Maximov M. (2021). III–V microdisk/microring resonators and injection microlasers. J. Phys. D: Appl. Phys..

[12] Zhang S., Li Y., Hu P. (2020). Unidirectional emission of GaN-based eccentric microring laser with low threshold. Opt. Express.

[13] Thubthimthong B., Sasaki T., Hane K. (2018). Electro-optic guided-mode resonance tuning suppressible by optically induced screening in a vertically coupled hybrid GaN/Si microring resonator. Appl. Phys. Lett..

[14] Zhang S., Li Y., Hu P. (2021). Realization of directional single-mode lasing by a GaN-based warped microring. Photonics Res..

[15] Kneissl M., Teepe M., Miyashita N., Johnson N. M., Chern G. D., Chang R. K. (2004). Current-injection spiral-shaped microcavity disk laser diodes with unidirectional emission. Appl. Phys. Lett..

[16] Chern G. D., Tureci H. E., Stone A. D., Chang R. K., Kneissl M., Johnson N. M. (2003). Unidirectional lasing from InGaN multiple-quantum-well spiral-shaped micropillars. Appl. Phys. Lett..

[17] Zhu G., Qin F., Guo J., Xu C., Wang Y. (2017). Unidirectional ultraviolet whispering gallery mode lasing from floating asymmetric circle GaN microdisk. Appl. Phys. Lett..

[18] Wang Q. J., Yan C., Yu N. (2010). Whispering-gallery mode resonators for highly unidirectional laser action. Proc. Natl. Acad. Sci. U. S. A..

[19] Yang Y. D., Zhang Y., Huang Y. Z., Poon A. W. (2014). Direct-modulated waveguide-coupled microspiral disk lasers with spatially selective injection for on-chip optical interconnects. Opt. Express.

[20] To C. H., Fu W. Y., Li K. H., Cheung Y. F., Choi H. W. (2020). GaN microdisk with direct coupled waveguide for unidirectional whispering-gallery mode emission. Opt. Lett..

[21] Liu D., Hattori H. T., Fu L., Tan H. H., Jagadish C. (2010). Increasing the coupling efficiency of a microdisk laser to waveguides by using well designed spiral structures. J. Appl. Phys..

[22] Jiang Y., Liu P., Wang Y. (2022). Experimental demonstration and theoretical analysis of simultaneous emission-detection phenomenon. ACS Omega.

[23] Li J., Wu J., Chen L. (2021). On-chip integration of III-Nitride flip-chip light-emitting diodes with photodetectors. J. Lightwave Technol..

[24] Yang Y., Zhu B., Shi Z. (2017). Multi-dimensional spatial light communication made with on-chip InGaN photonic integration. Opt. Mater..

[25] Yuan J., Gao X., Yang Y. (2017). GaN directional couplers for on-chip optical interconnect. Semicond. Sci. Technol..

[26] Feng M., Wang J., Zhou R. (2018). On-chip integration of GaN-based laser, modulator, and photodetector grown on Si. IEEE J. Sel. Top. Quantum Electron..

[27] Sun Y., Zhou K., Sun Q. (2016). Room-temperature continuous-wave electrically injected InGaN-based laser directly grown on Si. Nat. Photonics.

[28] Sun Y., Zhou K., Feng M. (2018). Room-temperature continuous-wave electrically pumped InGaN/GaN quantum well blue laser diode directly grown on Si. *Light Sci. Appl.*.

[29] Sun Q., Yan W., Feng M. (2016). GaN-on-Si blue/white LEDs: epitaxy, chip, and package. J. Semicond..

[30] Wang J., Feng M., Zhou R. (2019). GaN-based ultraviolet microdisk laser diode grown on Si. Photonics Res..

[31] Wang D., Zhu T., Oliver R. A. (2018). Ultra-low-threshold InGaN/GaN quantum dot micro-ring lasers. Opt. Lett..

[32] Zhang X., Li Z., Zhang Y. (2022). Heterogeneously integrated InGaN-based green microdisk light-emitters on Si (100). Opt. Express.

[33] Wang J., Hua Q., Sha W. (2022). Flexible GaN-based microscale light-emitting diodes with a batch transfer by wet etching. Opt. Lett..

[34] Xu H., Wright J. B., Hurtado A. (2012). Gold substrate-induced single-mode lasing of GaN nanowires. Appl. Phys. Lett..

[35] Xu H., Hurtado A., Wright J. B. (2014). Polarization control in GaN nanowire lasers. Opt. Express.

[36] Berini P., De Leon I. (2011). Surface plasmon–polariton amplifiers and lasers. Nat. Photonics.

[37] Jiang D., Liu B., Tao T. (2020). The optimization of surface plasmon coupling efficiency in InGaN/GaN nanowire based nanolasers. Appl. Phys. Express.

